# A technological framework for running and analyzing animal head turning experiments

**DOI:** 10.3758/s13428-017-0934-2

**Published:** 2017-07-14

**Authors:** Jinook Oh, Marisa Hoeschele, Stephan A. Reber, Vedrana Šlipogor, Thomas Bugnyar, W. Tecumseh Fitch

**Affiliations:** 1 Department of Cognitive Biology, University of Vienna, Althanstrasse 14, 1090 Vienna, Austria; 2 School of Life Sciences, University of Lincoln, Brayford Pool, Lincoln, LN6 7TS UK

**Keywords:** Head turn, Computer-aided video analysis, Computational ethology, Common marmoset, Animal cognition

## Abstract

Head turning experiments are widely used to test the cognition of both human infants and non-human animal species. Monitoring head turns allows researchers to non-invasively assess attention to acoustic or visual stimuli. In the majority of head turning experiments, the head direction analyses have been accomplished manually, which is extremely labor intensive and can be affected by subjectivity or other human errors and limitations. In the current study, we introduce an open-source computer program for measuring head directions of freely moving animals including common marmoset monkeys (*Callithrix jacchus*), American alligators (*Alligator mississippiensis*), and Mongolian gerbils (*Meriones unguiculatus*) to reduce human effort and time in video coding. We also illustrate an exemplary framework for an animal head turning experiment with common marmoset monkeys. This framework incorporates computer-aided processes of data acquisition, preprocessing, and analysis using the aforementioned software and additional open-source software and hardware.

## Introduction

The head turning experiment was first used by Dix and Hallpike (1947), and Suzuki and Ogiba (1961) to assess auditory perception in children. This procedure has been widely used since then to test cognition of not only in infants, but also various animal species such as cotton-top tamarins (*Saguinus oedipus*) in Fitch and Hauser (2004), chimpanzees (*Pan troglodytes*) in Okamoto, Tanaka, and Tomonaga, (2004), and dogs (*Canis lupus familiaris*) in Siniscalchi et al (2010). Today, head turning experiments are one of the valuable standard tools for investigating cognitive functions of subjects who cannot understand or follow verbal or written instructions.

Several types of experiments, such as testing the ability to discriminate novel auditory stimuli or assessing an animal’s attention to a set of visual stimuli, have been conducted using head turning procedures. Although the procedures in these experiments can vary in details, the common core procedures involve recording the subject’s head orientation direction during the entire experiment and identifying the direction that the focal subject is facing at a given time from this recorded video. These core procedures are typically performed with manual coding based on visual observations in the vast majority of studies. Not only is manual coding inherently subjective, manual coding often has been conducted without any exact and objective threshold, and in many cases a clear methodological description of the procedure was not provided. In the cases where a description is provided, the analysis is typically qualitative rather than quantitative due to subjective human interpretation.

In the current study, we developed and tested coding software for analyzing head directions and an exemplary framework, which also incorporates technological data acquisition and data preprocessing. Our two central aims were to increase efficiency and to increase objectivity. Regarding efficiency, we aimed to save time while increasing accuracy: scoring video of animal behavior manually is extremely time consuming in general, and this is particularly true if one attempts to analyze each frame of a video to get accurate temporal information (e.g., how long after a stimulus onset did the animal respond with a head turn or the total duration of stimulus looking time during an entire trial). A semi-automated system, which requires a bit of human input when the algorithmic result is inaccurate, but runs automatically otherwise, can provide high spatial and temporal accuracy with little human effort. Perhaps more importantly, regarding objectivity we began developing our framework in response to worries about how unconscious bias could enter into data collection and analysis in a behavioral experiment, which has become a major topic in animal cognition in particular (due in part to the allegations of bias surrounding the work of Marc Hauser at Harvard, Gewin (2012) and Hauser, Weiss & Marcus (2002)), as well as an important aspect of the replicability crisis in psychology more generally (Pashler & Wagenmakers 2012; Cesario 2014; Stroebe and Strack 2014; Simons 2014). In general, we believe that while increasing efficiency is highly desirable, increasing objectivity and accuracy is a crucial prerequisite for solid empirical work involving videos of animal behavior. We thus think that a good, low-cost solution for semi-automated coding will be of great value to many researchers in animal cognition and communication research, as well as for human research in some cases.

One potential alternative solution to the problems of efficiency and objectivity is to use eye-tracking, which is widely done for adult humans and increasingly attempted with animals (Senju & Csibra, 2008; Kimmel, Mammo, & Newsome 2012). Unfortunately, there are serious issues for performing non-invasive eye-tracking in unrestrained animals, that include issues with calibration (which can be done in animals but takes enormous training) and drift caused by postural readjustments during a series of trials. Thus, most published eye-tracking work on primates involves monkeys with surgically implanted eye coils (little calibration needed (Robinson 1963)) and restrained, often head-fixed animals (e.g., Bruce & Goldber, 1985; Groh et al., 2001). This, too, takes considerable training for animals to be comfortable. For many species and problems, this solution is simply untenable. Thus, our goal is to have a non-invasive solution, requiring little or no training of the animal subject, which can be broadly applied to free-moving animals.

The developed software is open source and available at http://www.github.com/jinook0707/HDC (Head direction coding software) and http://www.github.com/jinook0707/AHTE (scripts used in our common marmoset case). All of the open-source Python packages and programs used in this study are listed in Table 1.

**Table 1:** External packages and programs used by the experimental software

Software	Version	Reference
numpy	1.8	(Van der Walt, Colbert, & Varoquaux, 2011)
scipy	0.14	(Jones, Oliphant, Peterson, et al., 2001)
OpenCV	2.4	(Bradski 2000)
pyaudio	0.2	(Pyaudio 2006)
pyserial	2.7	(Pyserial 2001)
wxPython	2.9	(Wxpython 1998)
arduino	1.5	(Arduino Software 2014)
FFmpeg	1.1	(Ffmpeg 2000)

## Head direction coding software

Manual coding of head directions of an animal subject with recorded video data is labor intensive. Implementing software to automatically track animal head direction can be quite simple, if we attach visual markers to the subject’s head. For our American alligator case (one of the following subsections), we indeed tested using a color tag because applying a color tag on these reptiles is feasible due to the physical characteristics of the reptile skin. However, the software we introduce in this study does not require such artificial visual markers on animal subjects. We pursued an approach that did not require visual markers because attaching markers can be difficult depending on the type of animal and stress levels of animals can be elevated by the attachment and/or presence of the markers. Also, in our marmoset monkey case, any attached tag on the head could be easily removed manually by the monkeys. We also did not consider painted or sprayed color marking on their fur due to possible effects of artificial coloration on their social interactions.

Implementing entirely automatic tracking software without visual markings is possible, although it is not an easy task due to the highly deformable body structure of animals, especially in mammals and birds. One method would be to make software with machine learning algorithms, which would be costly in time for implementation and preparing sample pictures for training the learning algorithms. Furthermore, it would work only for one or a few specific species or body types.

Another method would be to implement semi-automatic software with relatively simple algorithms for one or several species, expecting somewhat more erroneous output data, but with less effort and shorter time in software implementation, compared to implementing machine learning algorithms.

In this study, we selected the latter method. Even though the second method could be less accurate, realistically neither will be accurate enough to avoid human validation. Furthermore, the latter method with manual input features can be easily adjusted to different animal species due to its simplicity. The software tool (Head Direction Coding software or HDC hereafter) that we developed for this study calculates head directions using a rather simple and straightforward algorithm, however, it accepts manual override input on any frame whenever a human coder deems it necessary. The method for manual input is simple and straightforward as well.

**Fig. 1:**
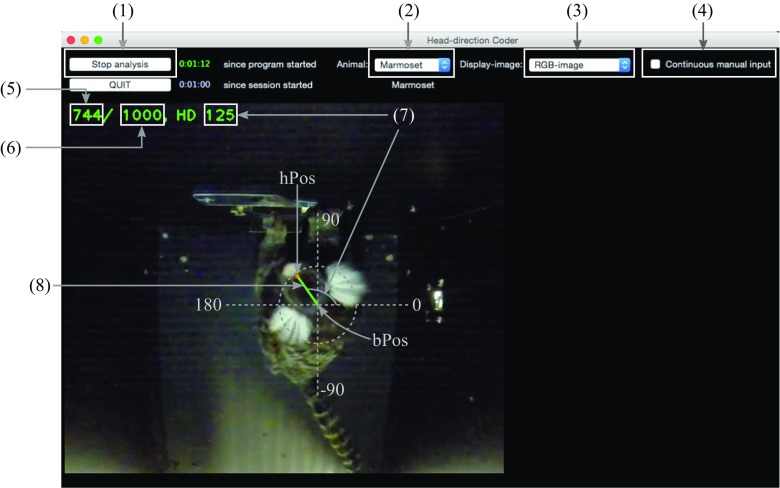
Screenshot of HDC; (1) Start/Stop analysis, (2) Determine species-specific algorithm, (3) Display different stages of image processing (for debugging purpose), (4) Toggle continuous manual input, (5) Current frame index, (6) Number of frames, (7) Head direction of the current frame, (8) Line graphically representing head direction (the *small circle* at one end of the line denotes *hPos* and the other end of the line is *bPos*)

**Fig. 2:**
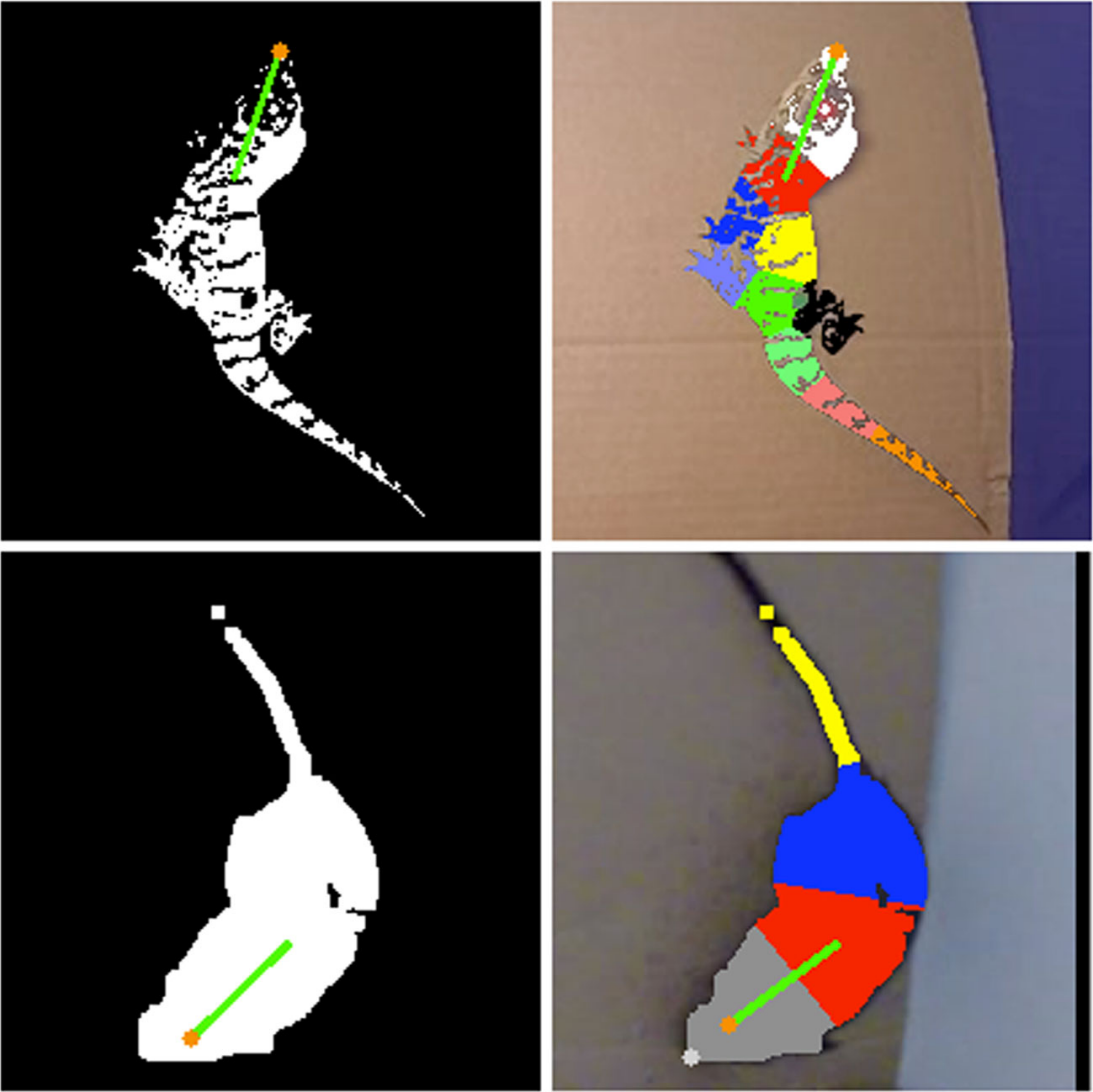
Alligator and gerbil binary image (obtained by subtracting background image) and clustered body points (by k-means clustering white points of the binary image)

**Fig. 3:**
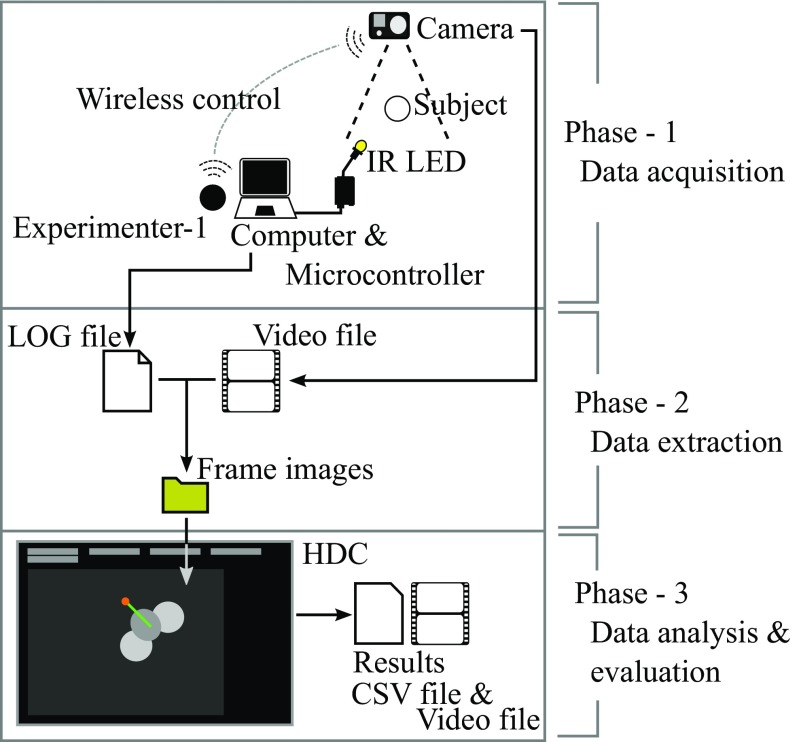
Framework overview to obtain the final CSV and video file data from the acquired experimental data of a common marmoset monkey

**Fig. 4:**
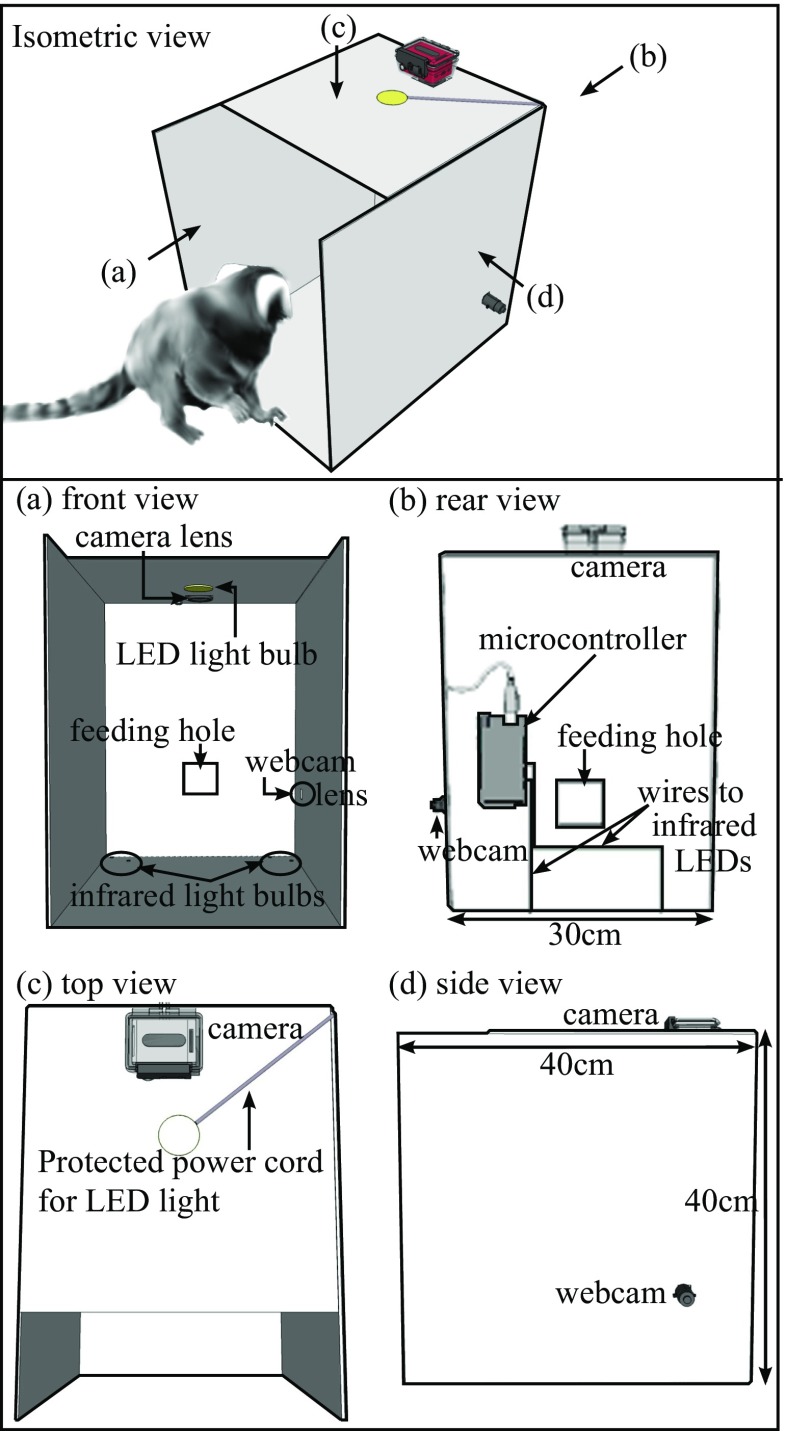
Experimental apparatus to record video of a common marmoset monkey’s head turning behaviors

HDC takes frame images (JPEG, PNG, or TIFF) as input data. When a frame image is loaded into HDC, a head direction for the loaded frame can be determined by a species-specific algorithm (this will be described in each species’ section) or manual input (a mouse click and drag on the loaded frame image). HDC generates a CSV file (see Table 2) and a video file as output files. The *mHPos*, manual *hPos* positioning, in the CSV file is a Boolean value to indicate whether a head position was determined manually and *mHD*, the manual head direction, in the CSV file indicates whether a head direction was determined manually. The difference between *mHPos* and *mHD* is that *mHPos* is True only when a mouse click and drag occurred, while *mHD* is True both on a manual input and while ‘continuous manual input’ (Fig. 1-(4)) is on. More descriptions about ‘continuous manual input’ will be given in the next subsection. The output video file is a record of analysis process in original video’s quarter frame size for a “sanity check” of coding performance.

**Table 2:** Example output CSV file hPos: position of anterior end of head, mHPos: manual hPos positioning, bPos: base position of a head direction line such as head center or neck, hDir: head direction, mHD: manual head direction

Frame-index	hPosX	hPosY	mHPos	bPosX	bPosY	hDir	mHD
1	946	250	True	923	254	9	True
2	926	248	False	897	253	8	False
3	930	248	False	901	253	8	False
4	930	248	False	901	253	8	False
...							
999	343	374	False	373	381	167	False
1000	346	372	False	375	381	164	False

### General program features

**Fig. 5:**
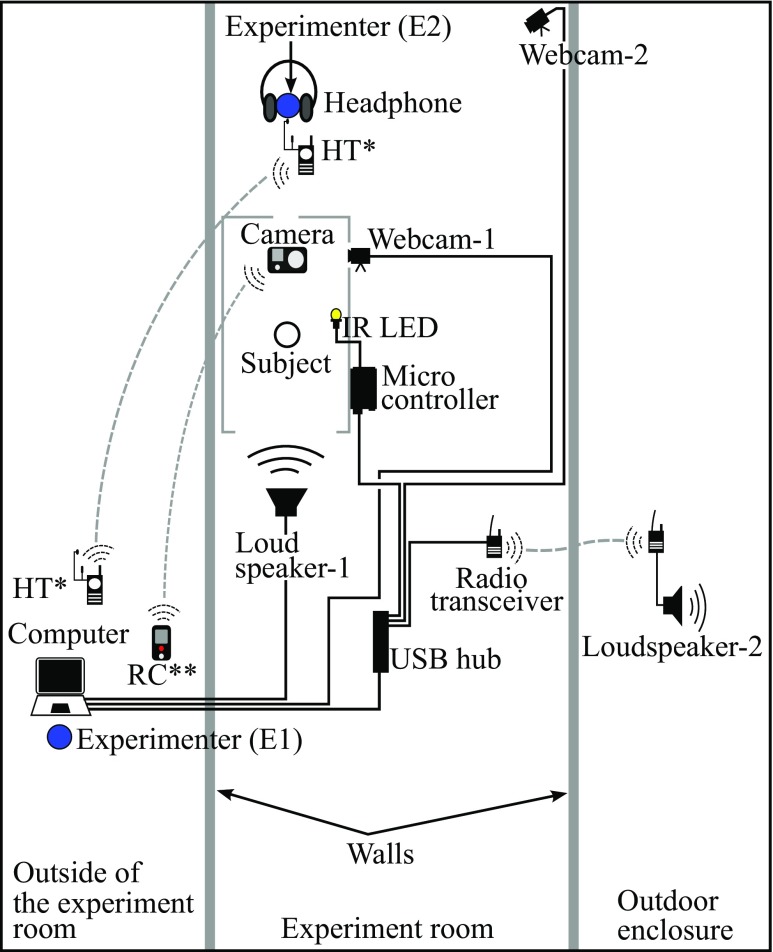
Schematic drawing of the electronic device setup for the head turning experiment. The *solid black lines* between electronic devices denote the connections via cables and the *dotted grey lines* denote the wireless connections.** HT: Handheld Transceiver with ear piece and microphone.*** RC: Remote control for the camera

**Fig. 6:**
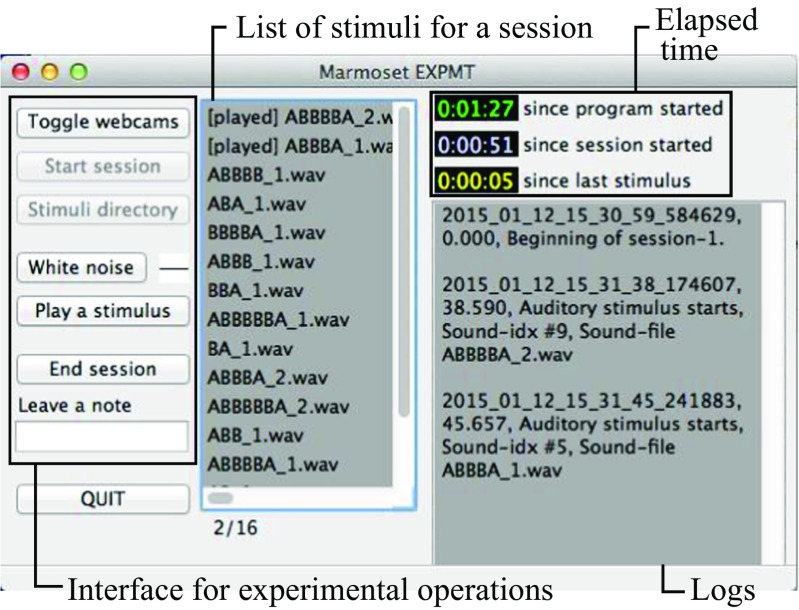
Screenshot of the experimental software. The software is to help an experimenter to start/end experimental sessions, watch the experimental room situation via webcams, play stimuli and leave notes in the log file

**Fig. 7:**
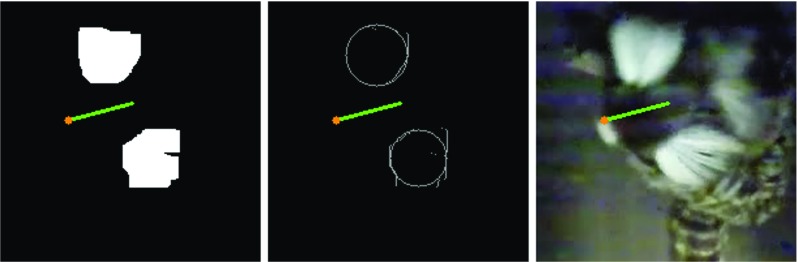
An example binary image, detected edge and result image of marmoset monkey head direction

**Fig. 8:**
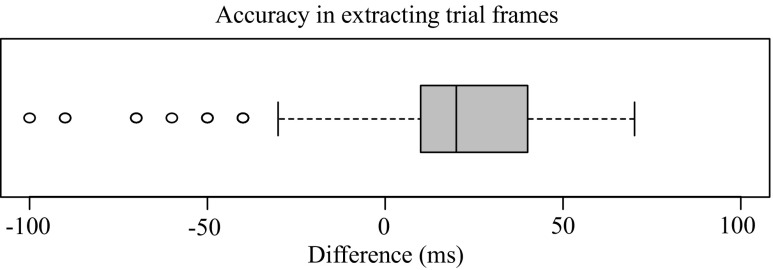
Accuracy in automatic extraction of trial frames. Difference is in milliseconds. The difference was obtained by counting how many frames the LED light emission frame was off from the 500th frame and multiplying by 10 (because each frame was taken every 10 milliseconds)

At the beginning of an analysis, a user specifies a folder that contains all of the frame images by pressing the ‘Start analysis’ button (Fig. 1-(1)). Filenames of the frame images must be formatted as ‘f*n*.jpg’ where *n* is zero padded six digits representing the frame number; it starts with a letter ‘f’ and the file extension is ‘jpg’. This format string can be changed in the *init* function of *HDCFrame* in *hdc.py* file. The selected folder should also have ‘bg.jpg’ file, which is a background image showing the scene without an animal subject. A user can navigate forward or backward through frame images with the right and left arrow keys. An arrow key can be combined with the Shift or Ctrl (Cmd in OSX) key to increase the number of frames it navigates to 10 or 100 frames, respectively. The ‘Spacebar’ key is used to toggle on/off continuous analysis of frames. In HDC, analysis and storing of the resultant data (including the head direction) is conducted as navigation through each frame occurs. The ‘M’ key or the ‘Continuous manual input’ checkbox (Fig. 1-(4)) is used to toggle on/off continuous manual input. A user can pause analysis by pressing the spacebar at any frame and manually set a head direction (Fig. 1-(8)) with a mouse click and drag. When ‘continuous manual input’ checkbox is checked, the manually entered head direction will be kept in following frames regardless of algorithms until the user turns it off by clicking the checkbox again. This is useful when the animal’s behavior causes an error in the applied algorithm for prolonged time. Such confusing behaviors include standing up with two legs, curling its body, twisting its head, which makes the target feature to disappear from the scene and so on. When no manual input is given, the current frame image is processed (see general image processing section below), then passed to a species-specific function to obtain *hPos*, *bPos*, and the head direction.

### General image processing

This subsection describes what HDC calculates from a frame image before it applies the species-specific algorithm to the image. It is composed of three processes: (1) first, obtain a binary image of the animal body, (2) then gather edge information using an edge detection algorithm on the obtained binary image, and (3) detect motion, to trigger a species-specific algorithm.

To obtain a binary image of the animal body, HDC first calculates absolute differences between a frame image and the stored background image, *I*_*i*_ = |*F*_*i*_ − *B* *G*_*i*_| where *i* is a pixel index of x, y, and color channel. This image of differences is converted to a greyscale image. Then, noise, small differences, and even small body parts depending on a user-defined parameter, *mExOIter*, are deleted with OpenCV’s function, ‘morphologyEx’ (Bradski and Kaehler 2008, p. 136), which can be written as the following formula, *I*_*i*_ = max((min(*I*_*N*_))_*N*_) where *i* is a pixel index and *N* is a set of pixel indices including *i* and *i*’s eight neighboring pixels. The user-defined parameter, *mExOIter*, determines the number of iterations of this formula. This image is converted to a binary image using a user-defined threshold. The binary image can be displayed by changing ‘Display-image’ (Fig. 1-(3)) to ‘Greyscale(Diff)’.

The binary image is then processed with the Canny edge detection algorithm (Canny 1986). The detected edges can be displayed by changing ’Display-image’ (Fig. 1-(3)) to ‘Greyscale(Edge)’. The minimum bounding rectangle (MBR hereafter) of all the contours and each contour’s size and center points are calculated with the obtained contours from the Canny algorithm. MBR is a minimum rectangle which can bound all the given points, which can be expressed (*x*, *y*, *w*, *h*) or (*x*1, *y*1, *x*2, *y*2) where (*x*, *y*) represent upper left corner, *w* is width, *h* is height, (*x*1, *y*1) represent the upper left corner and (*x*2, *y*2) represent the lower right corner.

Finally, HDC checks whether there was motion or not by calculating the absolute difference (absdiff) between the current frame image and the last frame in which motion was detected. Only when there is motion in the frame ($\theta _{l} \leq \sqrt {{\sum }_{x,y}{absdiff} / 255} < \theta _{u}$, while *𝜃*_*l*_ and *𝜃*_*u*_ are lower and upper thresholds), it does pass the data to the species-specific function. Otherwise, the previous frame’s *hPos*, *bPos* and head direction are used.

### Species-specific parameters and processing

There are currently six species-specific parameters that are necessary for general image processing. They are *mExOIter* (number of iterations of the aforementioned morphologyEx), *thParam* (threshold to produce binary image), *contourTh* (minimum half girth of a contour; minimum width+height), *motionTh* (lower and upper thresholds to determine motion), *degTh* (maximum angle difference between previous and current head direction) and *hdLineLen* (length of head direction line). These parameters change when a user changes species (Fig. 1-(2)) and they are set in *onChangeAnimalSp* function in *hdc.py*.

The species-specific image processing is one function in HDC. This function is different depending on the target species’ body structure, features, colors, and so on. This function will be described for each animal test case.

## American alligator/Mongolian gerbil case

We tested HDC with the American alligator and Mongolian gerbil (Fig. 2). The alligator video used in this study originated from an unrelated study on early-life behavior in Alligatoridae conducted by Reber et al. at “Crocodiles of the World”, a zoo in the UK, in October 2015 (manuscript in preparation). The study was given ethical approval by the Faculty Research Committee of the University of Lincoln, UK in April 2015 (reference number: CoSREC36). Gerbil videos were recordings of a pet of a coauthor (TB) made specifically to test HDC. Both videos were recorded at 60 frames per second (FPS) with a video camera (Hero3+ Black edition, GoPro, USA).

An algorithm for the American alligator was implemented first. The same algorithm was also successful for Mongolian gerbil with slightly different user parameters such as *k* for k-means algorithm and *mExOIter*.

### Species-specific algorithm

First, this algorithm determined *hpt*, which was a projected point based on a previous head direction calculated as (*b*_*x*_ + *c* *o* *s*(*d*) ⋅ *l*, *b*_*y*_ − *s* *i* *n*(*d*) ⋅ *l*) where *b* was previous *bPos*, *d* was previous head direction, *l* was the longer length between width and height of MBR of all contours. White points in the binary image from general image processing were clustered using a k-means algorithm and an additional species-specific parameter *k*. The *k* was set to 10 for American alligator and four for Mongolian gerbil. When more body parts are visible (more complex body shape), the algorithm was more successful with larger *k*. Distances between *hpt* and each cluster’s centroid were calculated. The closest cluster to *hpt* was assigned to a head cluster and the closest point to *hpt* in the head cluster was determined as *hPos*. Distances between the head cluster’s centroid and each of other cluster’s centroids were calculated. The centroid of the closest cluster to the head cluster was determined as *bPos*. The angle of the line connecting *hPos* and *bPos* was calculated as *atan2* (−(*h*_*y*_ − *b*_*y*_),(*h*_*x*_ − *b*_*x*_)) where *atan2* is an arctangent function for appropriate quadrant output, *h* is *hPos*, *b* is *bPos*. This angle was determined as a head direction. Example results are shown in Fig. 2.

### Additional test with a color tag

Although the tested alligator hatchling had a plastic color tag on the center of its head and the base of its tail, these tags were not used in the above species-specific algorithm. We additionally tested using the color tag on the head. In this test, the algorithm was the same as the above species-specific algorithm except that *bPos* was determined using a color detection algorithm on the color tag as follows. The frame image was converted (Smith 1978) to HSV (Hue, Saturation and Value) from RGB (red, green and blue) format. The red color was detected using OpenCV’s ‘inRangeS’ function (Bradski and Kaehler 2008, p. 65) with a range of (175,100,90)-(180,255,255). The position of red color tag was calculated using OpenCV’s ‘Moments’ (Bradski and Kaehler 2008, p. 252) as (*m*_10_/*m*_00_, *m*_01_/*m*_00_) and determined as *bPos*. This approach reduced errors in confusing situations such as an alligator turning sharply to one direction so that its head touches its fore limb. As it was already discussed in ‘Head direction coding software’ section, a conspicuous color tag or marking will obviously make the tracking task easier, but only when the target species can accept it physically and behaviorally (such as this reptile species).

## Common marmoset case

The alligator and gerbil cases illustrate the applicability of HDC’s features, assuming that the goal is only to analyze a resultant videos of an experiment. In the common marmoset case discussed in this section, we present an entire cognitive experiment where assessing head turning was important. This experiment was designed and conducted with many considerations in mind, including reduction of experimenter’s bias, errors, and time in data extraction and analysis with a semi-automatic coding program. We think this is an exemplary case to set a standard for experiments concerning an animal’s head direction, hence more details will be described in this section, and the software and hardware used introduced.

### Ethics

The housing conditions and the experimental design were in accordance with Austrian legislation and the European Association of Zoos and Aquaria (EAZA) husbandry guidelines for *Callitrichidae*. The research complied with protocols approved by the Institutional Review Board for animal experimentation (license number 2015-012) and adhered to all legal requirements in Austria.

### Framework overview

The experimental framework in this case study involves three phases, namely data acquisition, data extraction, and data analysis as shown in Fig. 3. During the data acquisition phase, an experimenter uses custom software to control many aspects of the experiment. In the data extraction phase, trial single frame images are extracted from a session video file based upon LED light signals from the first phase. In the analysis phase, a head orientation direction for each extracted frame is calculated using HDC (as described above).

### Preparation of experimental hardware and software

#### Experimental apparatus

The experimental apparatus (Fig. 4) was positioned inside a larger experimental cage. One side of the apparatus was completely open, therefore the monkey subject could freely move in and out of the apparatus within the experimental cage. During each trial, the subject was fed with preferred food items by an experimenter through a feeding hole (see Fig. 5) on the side opposite the entrance of the apparatus to entice the monkey into the apparatus and orient it away from a loudspeaker (AV-40, M-AUDIO, USA). A video camera (Hero3+ Black edition, GoPro, USA) with a high-speed recording capability situated above the apparatus continuously recorded the entire experimental session at 100 FPS. Additional video from a webcam on the side wall was used by an experimenter (see Fig. 5) to monitor the monkey inside the apparatus during the session. The video from this webcam was not recorded. Below, ‘camera’ will always refer to the recording camera on top of the apparatus, not the monitoring webcam. An LED light on the ceiling of the apparatus brightened the inside of the apparatus, and infrared LED light bulbs on the bottom, controlled by a microcontroller (Arduino UNO, Arduino, Italy), were used to emit light indicating the beginning of a session and onset of each stimulus play. The light emission at the start of a session was used to synchronize the video recorded by the camera with the time recorded in the session log file. A custom program to control and monitor experimental sessions (‘ExCon’) generated the session log file. The light emission marking the beginning of each stimulus play was used to measure accuracy of automatic extraction for trial frame images.

#### Other experimental hardware

Before starting a session, electronic systems were arranged as in Fig. 5. The computer was used by E1 to control the overall experimental procedure using ExCon. Two handheld transceivers (M48 plus, CTE International, Italy) were used to enable verbal communication between two experimenters. A remote control was used to start or stop the video recording of the camera. Over-ear headphones played loud music to E2 to mask the playback stimuli and avoid any unconscious cueing. Webcam-1 was used to monitor the inside of the apparatus, while webcam-2 was used to monitor the entire experimental cage. Loudspeaker-1 was used to play acoustic stimuli to the marmoset in the experimental apparatus, while loudspeaker-2 was used to play masking white noise to other marmosets in an outdoor enclosure to prevent any pre-exposure to future subjects.

An automatic machine feeder instead of E2 was also considered, but we preferred a human in this position for several reasons. First, various types of food including wet food for coaxing were changed depending on the monkey’s reaction to the food during a session to keep the subject’s interest. Second, the human experimenter was very experienced with marmoset monkeys, and thus able to judge when to stop the session due to distress or lack of interest. Third, monkeys were familiar with the human experimenter, while a mechanical feeder’s appearance and motor noise can cause fear for some subjects, resulting in more difficult and longer apparatus habituation. However, depending on the specific animal experiment situation, an automatic machine feeder maybe preferred.

Because this experimenter, E2, listened to loud music throughout the experiment, she was unaware of the stimuli played and unable to bias the results.

#### Experimental control program

The function of the experiment running program, ExCon (See Fig. 6), was to load, randomize, and play acoustic stimuli, play white noise, monitor the experimental room via webcams, send signals to a microcontroller to turn on/off infrared LED lights, and log all the important operations with timestamps.

### Data acquisition

To avoid human bias such as unconscious cueing, two experimenters were involved in running the experiment. E1 conducted the experimental session with ExCon, a remote control for video recording, and a handheld transceiver. E2, who was always unaware of the stimuli being presented, coaxed the monkey into the experimental chamber and fed the monkey subject following E1’s instructions, which were to feed or stop feeding.

An experimental session was conducted as follows. A monkey subject was moved into the experimental cage and all other marmosets were moved to the outdoor enclosure. E1 then started a session by commencing video recording via a remote control, and then clicking on the ‘start session’ button of ExCon. During each trial, E1 waited until the monkey was attending to the feeding hole, facing toward E2, to eat the food provided through the hole, E1 then clicked the ‘white noise’ button. A white noise stimulus, which was 10 s long, was then played to the other marmosets in the outdoor enclosure via loudspeaker-2. If the monkey was still attending to the food during the white noise, E1 directed E2 to stop feeding, clicked ‘play a stimulus’ to play an auditory stimulus, which was 0.42–1.44 s long, via loudspeaker-1 and waited for at least 15 s before starting the next trial. E1 conducted 16 trials per session.

### Data extraction

The majority of the resultant video was irrelevant data because the head turning behavior was important only during a short time period before and after stimulus playback. Therefore, the next step was to extract the relevant data (frame images of each trial) for the head direction calculation step, using the LED light emissions described previously. ExCon turned on the LEDs as soon as the experimenter clicked ‘Start session’ button. The time difference between the start of the session and the initial light emission in the resultant video showed how long the timestamps in ExCon’s log file are delayed, compared to the time in the video. The relevant video data extraction points were determined by searching for the initial LED light emission, calculating the delay time, and adding the delay time to the timestamps of each stimulus play in ExCon’s log file.

Each frame image of the video file was converted to HSV format from RGB format. The color detection for white color in the minimum bounding rectangle for the LED light bulbs of the first frame image was executed with OpenCV’s ‘inRangeS’ function.

The zeroth image moment (*m*_00_), hereafter denoted simply as ‘*m*’, of the output greyscale image of ‘inRangeS’ function was calculated with OpenCV’s ‘Moments’ and stored in a variable. This is essentially just the total sum of pixel intensity, scaled by 255. This image moment, *m*, was calculated for each consecutive frame until the difference between the initial *m* and the current *m* was greater than a threshold. The threshold was heuristically determined and remained the same for all sessions. The number of frames required to reach this difference of *m* was used to calculate the time difference, Δ*T*, in milliseconds between the video recording initiation and the experimental program’s session initiation. This calculated time difference was inserted at the beginning of the session log file. Then, another Python script used this time difference and the stimulus onset time of each trial recorded in the log file to extract the frames that made up each trial using FFmpeg. This resulted in ten seconds of frames from the video: 5 s before the stimulus onset frame and 5 s after the stimulus onset frame (this time span can be easily adjusted). These frames were cropped so that only the required center area remained, in which the monkey subject could appear. The result frame images from the above extraction process for each trial were stored in a folder, programmatically generated with a name including the group number, subject’s name, trial number, and the presented stimulus type.

### Data analysis and evaluation

This analysis was conducted using HDC with a marmoset-specific algorithm. A result example is in Fig. 7. In general image processing, most body parts except two white ear tufts were excluded by setting *mExOIter* (number of iterations of morphologyEx) with a high value, eight. Contour information, each contour’s size and center point, was calculated after the exclusion. The two largest contours of these contours were assigned to ear contours. The middle point of a line connecting the two ear contours was determined as *bPos*. This ear connecting line was then rotated by 90 ^∘^, one end point of the rotated line, whichever made the resultant head direction closer to the previous head direction, became *hPos*. The head direction vector was then calculated with *hPos* and *bPos* in the same way as the alligator/gerbil case.

### Results

Experimental data from 128 trials with eight different monkey subjects were processed computationally. Although a total of 128 000 data entries were generated through data analysis, only 128 assessments of head turning occurrences (Boolean values) were required for this specific experiment. However, more exact and demanding data assessments are often required such as precise head angle, or assessing length of time facing a specific direction. In such a case, all 128 000 data assessments could be required, which would have taken significantly more human effort and time if coded in a completely manual way. A comparison between manual and semi-automatic method will be described in the next section.

#### Extraction of trial frames

How accurately the trial frames were extracted from the method described in the ‘Data extraction’ section is depicted in Fig. 8.

The time difference was measured by human visual observation of LED light emission for stimulus onset, which should be observed in the 500th frame in the 1,000 extracted frame images of each trial, if the frame extraction procedure was accurate. The video data and the log file’s timestamp were synchronized once for the start of a session using computer vision algorithms. However, onset of each trial stimulus was calculated by adding the calculated difference between the video start and session start to the timestamp of stimulus play within the log file. Therefore, Fig. 8 shows inaccuracies in generating timestamps in ExCon and/or latency in the microcontroller’s operations to receive a signal from ExCon and turn on LED lights.

These inaccuracies could have been eliminated by extracting trial frames using the LED light emission for each trial and the same algorithms that we used to determine the frame in which the session was started. However, this approach was not applied due to the increased amount of data processing time depending on the lengths of recorded video files. The inaccuracies from this approach are well within the tolerance range for assessing head turning experiment results.

### Head direction data

**Fig. 9:**
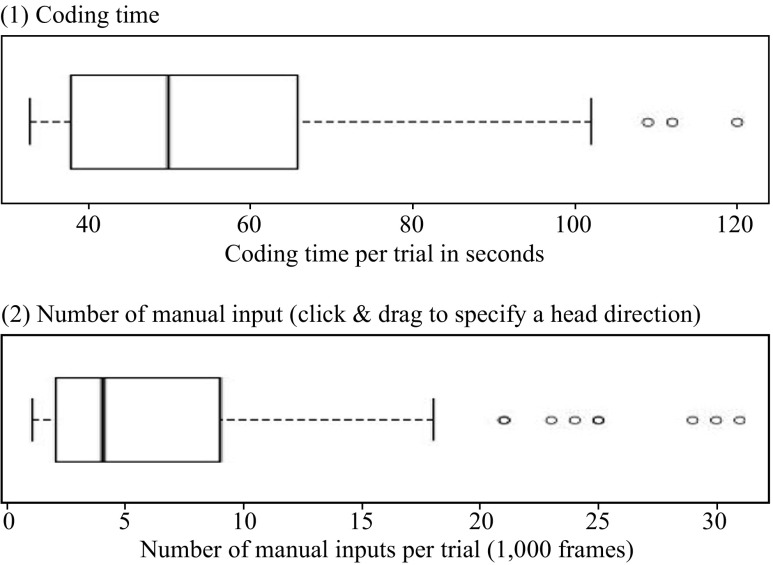
Elapsed time and number of manual inputs per trial in coding head directions

The final output of the analyzing software HDC was immediately viewed and corrected by a coder when its algorithm incorrectly analyzed head directions. Thus, the coder had to invest some time to produce the final data. Elapsed time for coding the head directions of video frames for 128 trials was about 117 min. Thus, the average time for coding was 54.74 s per trial (median: 50, SD: 20.354) as shown in Fig. 9-(1).

When the algorithm failed to correctly analyze the head direction, it was necessary for the user to manually specify the head direction by a mouse click and drag. The measurement of the number of manual input indicates the coder’s effort, and the efficiency of the analyzing software as well. In total, there were 861 frames with manual input which was about 0.673 % of all 128 000 frames. The average number of these manual inputs was 6.727 times per trial (median: 4, SD: 6.747) as shown in Fig. 9-(2).

## Comparison with manual coding

Whether electronics and computers with custom software are truly needed to conduct and analyze an experiment depends on several points such as the type of experiment, the subject species, and measurement resolution. We tested the analyzing software HDC with 3000 sample video frames of freely moving animals, the aforementioned three species, under an assumption that we need continuous measurements of the head direction. Three coders (JO, VS, and SR) coded 3000 frames, 1000 for each species, once with a completely manual method and again with the semi-automatic method already discussed. The manual method was conducted by indicating head direction using a mouse click and drag on each frame. The semi-automatic method is to determine head directions mainly with HDC’s algorithm, but to manually correct unacceptable algorithm output data.

The elapsed time of the manual method was about nine times longer than the semi-automatic method on average. In the semi-automatic method, the manual input was made on about 1 % of data on average as shown in Table 3.

**Table 3:** Human effort comparison of manual method and semi-automatic method; (ET: Elapsed Time in seconds, NM: Number of manual inputs, M: Manual method, SA: Semi-automatic method)

Coder	Species	ET (Manual)	ET (Semi–Automatic)	NM (M)	NM (SA)
JO	Marmoset	1135	71	885	10
	Alligator	1055	133	999	11
	Gerbil	1193	273	999	5
VS	Marmoset	1622	176	717	23
	Alligator	1438	188	990	9
	Gerbil	1426	241	825	1
SR	Marmoset	1542	83	846	2
	Alligator	1468	185	932	31
	Gerbil	1521	238	897	2

There were some frames in which a monkey was out of the video scene, hence the numbers of manual input in manual method, NM(M), of each coder were below 1000. However, NM(M) was supposed to be 1000 in alligator and gerbil videos because a subject was present on all 1000 frames in the video of each animal. The numbers less than 1000 in NM(M) in those two animal videos represent human error (missing some frames by accident).

**Fig. 10:**
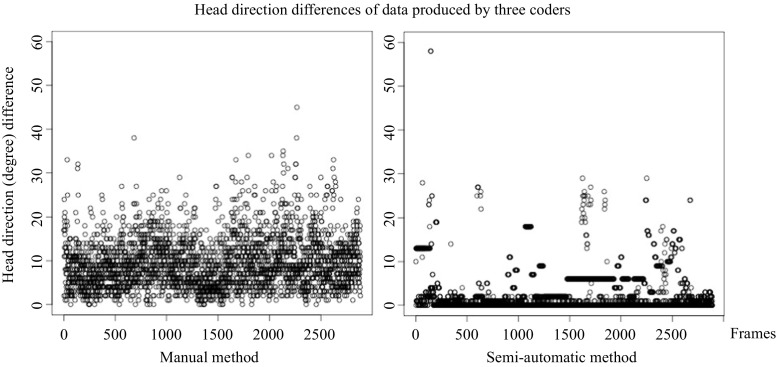
Head direction difference. Each *data point* represents the maximum absolute difference among the three head directions coded by three coders

For both methods, manual and semi-automatic, there was excellent agreement between the coders (ICC ≥ 0.955, F ≥ 22.077, *p* < 0.001). However, the variance among the coders was significantly greater in the manual method (Wilcoxon signed-rank test, *N* = 2883, *Z* = -8.211, *p* < 0.001, see Fig. 10).

## Conclusions

In the present study, we built semi-automatic head direction analysis software and established a complete technological framework, incorporating data acquisition, processing, and analysis, needed to perform and analyze a head turning experiment. With this framework, we were able to obtain a larger quantity of measurements with better accuracy, less human bias, and less human effort in a much shorter time, compared to manual coding of the same recorded videos.

By using computer vision algorithms, our framework has the advantage that it did not require restraining animals, which can cause an animal much higher stress levels than usual in its captive environment. We aimed to collect data without causing a high stress level, both for ethical reasons and because stress can alter cognitive functioning (McEwen and Sapolsky 1995; Mendl 1999). This may lead to more accurate assessment of normal cognitive functioning, and supports overall animal welfare.

Our video analyzing software, HDC, can be used as base software to analyze resultant video data of any animal head turning/gaze experiment with relatively small modifications (setting species-specific parameters and inserting one function for the target species). With a framework such as our example framework from the marmoset monkey head turning experiment, many different experiments looking at animal head directions in freely moving situations are possible.

Overall, our software and framework enabled us to increase our productivity and the quality of results and to reduce subjectivity in acquiring and analyzing data in an animal head turning experiment. We anticipate that such advantages offer important benefits for many researchers working in animal cognition.
